# SARS-CoV-2 serology in 4000 health care and administrative staff across seven sites in Lombardy, Italy

**DOI:** 10.1038/s41598-021-91773-4

**Published:** 2021-06-10

**Authors:** Maria Teresa Sandri, Elena Azzolini, Valter Torri, Sara Carloni, Chiara Pozzi, Michela Salvatici, Michele Tedeschi, Massimo Castoldi, Alberto Mantovani, Maria Rescigno

**Affiliations:** 1 IRCCS Humanitas Research Hospital, Rozzano, Milan, Italy; 2 Istituto Di Ricerche Farmacologiche, Mario Negri” - IRCCS, Milan, Italy; 3 Department of Biomedical Sciences, Humanitas University, Pieve Emanuele, Milan, Italy; 4 The William Harvey Research Institute, Queen Mary University of London, London, UK; 5 Humanitas Gavazzeni and Castelli, Bergamo, Italy

**Keywords:** Adaptive immunity, Biomarkers

## Abstract

Lombardy is the Italian region most affected by COVID-19. We tested the presence of plasma anti-SARS-CoV-2 IgG antibodies in 3985 employees across 7 healthcare facilities in areas of Lombardy with different exposure to the SARS-CoV-2 epidemic. Subjects filled a questionnaire to self-report on COVID-19 symptoms, comorbidities, smoking, regular or remote working, and the exposure to COVID-infected individuals. We show that the number of individuals exposed to the virus depended on the geographical location of the facility, ranging between 3 and 43%, consistent with the spatial variation of COVID-19 incidence in Lombardy, and correlated with family interactions. We observed a higher prevalence of females than males positive for IgG, however the level of antibodies was similar, suggesting a comparable magnitude of the anti-spike antibody response. IgG positivity among smokers was lower (7.4% vs 13.5%) although without difference in IgG plasma levels. We observed 11.9% of IgG positive asymptomatic individuals and another 23.1% with one or two symptoms. Interestingly, among the IgG positive population, 81.2% of subjects with anosmia/dysgeusia and fever were SARS-CoV-2 infected, indicating that these symptoms are strongly associated to COVID-19. In conclusion, the frequency of IgG positivity and SARS-CoV-2 infection is dependent on the geographical exposure to the virus and primarily to family rather than hospital exposure.

## Introduction

Lombardy has been the Italian region most affected by SARS-CoV-2 infection with more than 80,000 cases from the outbreak (as of May 8th 2020, https://utils.cedsdigital.it/coronavirus/#regioni1). Only symptomatic individuals have been tested by RT-PCR to detect SARS-CoV-2, and hence the spreading of the virus, particularly in the asymptomatic population, is unknown. There have been cases in which viral detection was missed, as for instance when patients with negative RT PCR test convert to positive in few days, or when COVID-19 is highly suspected by the result of chest CT^1–6^. It is just a snapshot of the exact moment of the infection. Hence, unless being continuously monitored by viral testing, the asymptomatic individuals’ infected state may be missed.

A retrospective way to assess viral spread is via the analysis of an immune response that can be detected through the development of immunoglobulins^7^. Indeed, a large share (> 95%) of laboratory-confirmed COVID-19 showed seroconversion even using different methodologies and antibody isotypes (ELISA, EIA, anti-IgM, anti-IgG or anti-IgA)^7–11^. Interestingly, the dynamic of the immune response to some viruses, including SARS-CoV-2, is very peculiar and patient dependent. IgM for instance, which should be the first type of immunoglobulins to appear under normal circumstances, are detected before, concomitant or even after IgGs, or do not appear at all^7,12^. Thus IgG is a more reliable marker for seroconversion particularly for past infections.

There is evidence that IgG analysis allowed to detect viral RNA negative asymptomatic relatives of COVID-19 patients^7^, suggesting that anti-SARS-CoV-2 IgG may be used as a diagnostic tool to detect the incidence of asymptomatic infections even in cases of impossibility of viral detection or long after viral clearance.

## Results

### Study population

Recruitment was on a voluntary basis: it started on April 28th 2020 and more than 65% of employees (n = 3985) participated as of May 16th 2020 (clinicaltrial.gov NCT04387929). The prevalence of COVID-19 was 9.7% in the studied population (386 out of 3985 subjects were affected by COVID-19). We tested the presence of plasma anti-SARS-CoV-2 IgG antibodies in nearly 4000 (3985) employees of 7 different healthcare facilities, one including a research center and a University, located across the Lombardy region in areas with different exposure to the epidemic (Milan, Rozzano, Varese, Castellanza and Bergamo, the city most affected by the pandemic per number of individuals). All of the analyzed hospitals drastically reduced their routine practice and, except for Humanitas Medical Care, which is an out-patient clinic, they were all dedicated to COVID-19 during the time of observation.

The individuals were healthcare professionals (physicians (16.5%), surgeons (7.2%) anesthesiologists (2.8%), physiotherapists (1.8%), nurses (25.4%)), technicians (4.4%), students (0.7%), researchers/academics (3.1%), other roles, including administrative staff (7.9%), biologists (1.3%). 66.8% were females and 33.2% males, median age 42 yo (21–86 yo) (Supplementary Table S1). The distribution of the healthcare workers participating to the study among the different hospitals was very similar except for HMC, an out-patient clinic, where the proportion of workers differs from that of the other hospitals, and for ICH which is the only site where researchers work (Supplementary Fig. S1). Besides this, we did not observe major differences in the voluntary participation of the different professionals across sites.

Subjects filled a questionnaire reporting whether they had COVID-19-associated symptoms (including fever, low grade fever, cough, sore throat and/or runny nose, asthenia, soreness and muscle pain, anosmia/dysgeusia, gastrointestinal symptoms, conjunctivitis, dyspnea, chest pain) and clinical manifestations as tachycardia and/or pneumonia (the latter confirmed by TC), from February 1st 2020 to the time of questionnaire filling. They also reported on body mass index (BMI), comorbidities (e.g. hypertension, heart disease or diabetes, immunosuppression), smoking, vaccinations (influenza, pneumococcus, tuberculosis (BCG) or other vaccines), regular or remote working, the exposure to COVID-19-infected individuals and whether they have been diagnosed with COVID-19. With the exception of HMC, which being an out-patient clinic closed down at lockdown (officially started on March 9th 2020), during the pandemic and in the period of observation, most of routine practice was interrupted, and the majority of physician and non-physician healthcare professionals worked in COVID-19-dedicated areas independent on the location of the hospital (for instance at ICH, 8 out of 20 wards were transformed into COVID-19 treatment wards (330 beds), and 2 into semi-intensive care units, while the intensive care units (ICU) capacity doubled (60 beds)). All of the personnel working in the emergency room or customer care had to wear obligatory PPE from February 1st 2020 at Castelli and Gavazzeni, from February 23rd 2020 at ICH, and from March 3rd 2020 everywhere in the hospital. Administrative staff workers stopped working in the hospital at the beginning of lockdown and worked from home. Customer service and research personnel worked one or two days a week at the sites and the rest of the time from home.

### Frequency of IgG positivity and correlation with geographical area

Anti-S1 and anti-S2 SARS-CoV-2 IgG specific antibodies were detected via an indirect chemiluminescence immunoassay. According to kit manufacturer, the test discriminates among negative (< 12AU/mL; with 3.8 as the limit of IgG detection), equivocal (12.0–15.0 AU/mL) and positive (> 15.0 AU/mL) subjects. Of 3985 enrolled subjects, 3462 (87%) were negative, 76 (2%) equivocal and 447 (11%) positive (which together with equivocal summed up to 523 (13%)) (Supplementary Table S1). We decided to group together equivocal and positive subjects as they behaved very similarly both as separate groups or as grouped together (Positive—IgG ≥ 12.0 AU/ml) in subsequent analyses (for instance in correlation with the number of symptoms, as shown in Supplementary Fig. S2). We cannot exclude inclusion of false positives.

**Figure 1:**
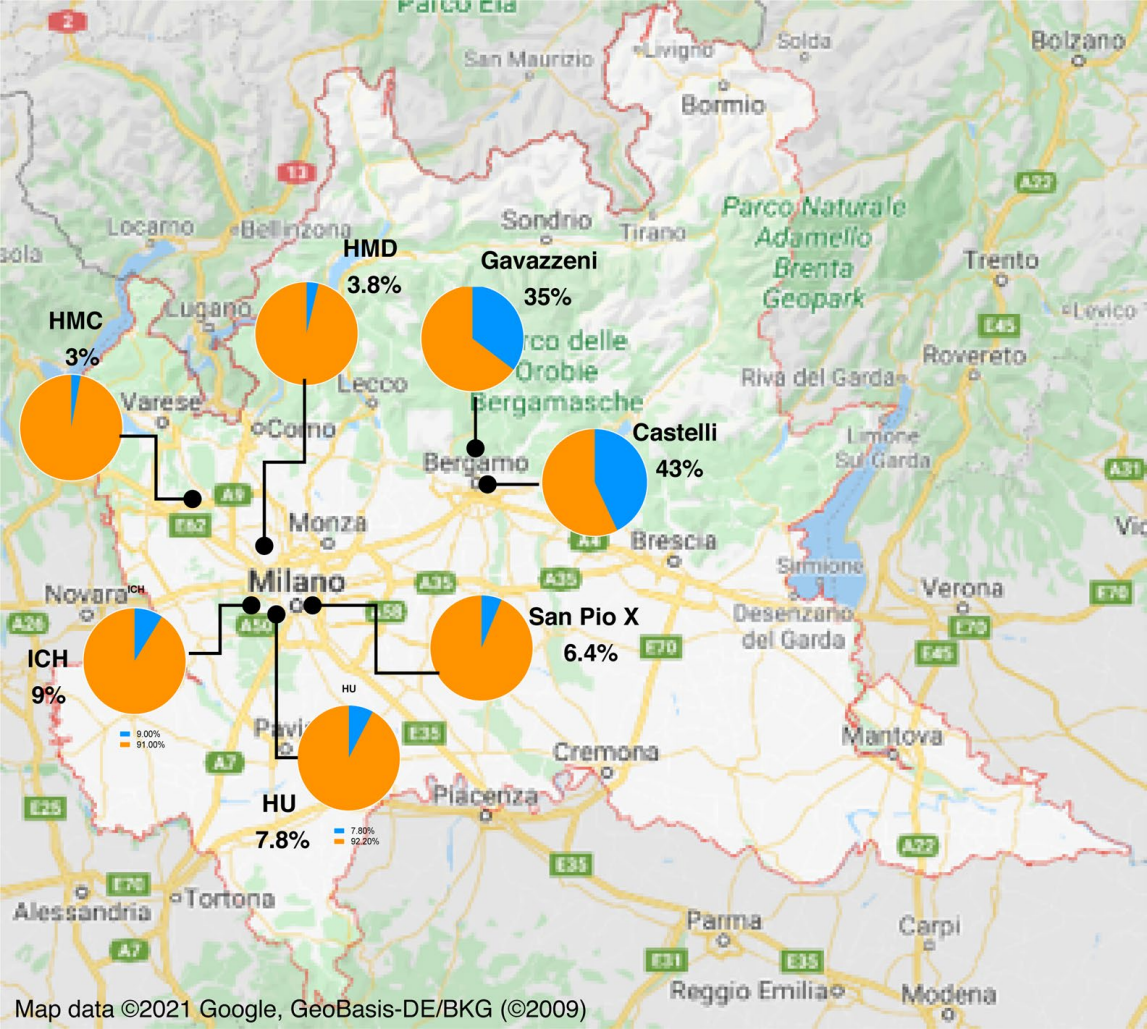
Distribution of IgG positivity (≥ 12 AU/mL) across different geographical areas and healthcare facilities in Lombardy, Italy. Employees from 7 different healthcare facilities were analyzed for their anti-SARS-CoV-2 IgG positivity. Pie charts show the percentage of negative subjects (IgG < 12 AU/mL) (orange) and that of positive subjects (IgG ≥ 12 AU/mL) (blue) in each site: Istituto Clinico Humanitas (ICH), Rozzano (MI); Humanitas Gavazzeni, Bergamo; Humanitas Castelli, Bergamo; Humanitas Mater Domini (HMD), Castellanza (VA); Humanitas Medical Center (HMC), Varese (VA); Humanitas University (HU), Pieve Emanuele (MI); Humanitas San Pio X, Milan, (MI). Satellite imagery has been modified from Google Maps (Map data 2021 Google, GeoBasis-DE/BKG (2009) Legal Notices https://www.google.com/intl/it_US/help/legalnotices_maps/).

The number of individuals exposed to the virus reflected the geographical area where the healthcare facility was located (Fig. 1). Two hospitals based in Bergamo (Humanitas Gavazzeni and Castelli) were the most affected as between 35 and 43% of the subjects was IgG positive. In the other sites the prevalence of IgG antibodies ranged between 3% (HMC) and 9% (ICH) (Supplementary Table S2). We applied a hierarchical (multilevel) model adjusting for clustering of individuals within healthcare facilities when assessing overall relationships between locations, professional status and the analyzed variables. Humanitas Gavazzeni in Bergamo was significantly different from all other facilities except for Castelli in Bergamo (Table 1 and Supplementary Fig. S3, *p* < 0.0001, OR and 95% CI indicated in the table). Interestingly, HMC and HMD which had the lowest incidence of IgG positive individuals (3.0% and 3.8%, respectively), are located in Varese, which is one of the less COVID-19 affected province in Lombardy (https://www.lombardianotizie.online/coronavirus-casi-lombardia/). In agreement, the percentage of patients hospitalized for COVID-19 was higher in Bergamo at Gavazzeni (56%), intermediate in Milan at ICH (36%) and low in Varese at HMD (10%) (not shown).

**Table 1:** Logistic analysis for geographical location of healthcare facilities.

Comparison	Odds ratio estimates	Lower 95% CI	Upper 95% CI	p-value
Humanitas Rozzano (ICH)	0.181	0.146	0.226	< .0001
Humanitas University (HU)	0.156	0.062	0.396	
Humanitas Medical Care (HMC)	0.057	0.014	0.234	
Humanitas San Pio X	0.126	0.074	0.215	
Humanitas Mater Domini (HMD)	0.073	0.041	0.131	
Humanitas Castelli	1.384	0.943	2.032	
Humanitas Gavazzeni—ref	1.00			

### Frequency of IgG positivity and correlation with gender, BMI and smoking

IgG positivity (≥ 12 AU/mL) was different between males (11.4%) and females (14%) (Supplementary Table S1), and, as shown in Table 2, this difference was retained in the multilevel logistic analysis (OR 0.73; 95% CI 0.57–0.94, *p* = 0.016). Thus, the observed effect was associated to a difference in gender susceptibility to infection or differences in exposure or to the ability to mount an antibody response rather than to a bias of the recruitment itself.

**Table 2:** Multilevel Logistic model for age, gender and their interaction, smoking habits, BMI.

Comparison	Odds ratio estimates	Lower 95% CI	Upper 95% CI	p-value
Physician vs other role	1.10	0.79	1.53	0.5039
Surgeon vs other role	1.10	0.71	1.71	
Anesthesiologist vs other role	0.49	0.22	1.08	
Physiotherapist vs other role	1.17	0.56	2.46	
Nurse vs other role	1.16	0.87	1.55	
Researcher vs other role	0.94	0.47	1.90	
Biologist vs other role	0.69	0.20	2.37	
Student vs other role	1.07	0.29	3.95	
Radiology technician vs other role	0.53	0.25	1.13	
Lab. technician vs other role	0.75	0.31	1.86	
Staff vs other role	1.04	0.67	1.61	
Smoking (number of cigarettes/day)	0.48	0.38	0.61	< .0001
Age > 60 vs ≤ 60	0.35	0.15	0.83	0.0166
Gender male vs female	0.73	0.57	0.94	0.0158
Interaction age*gender	2.98	1.08	8.21	0.0352
BMI	1.11	0.98	1.26	0.1127

Interestingly, 23.9% of subjects were smokers (n = 951) and IgG positivity among smokers was lower (7.4% vs 13.5%) (OR 0.45; 95% CI 0.34–0.60, *p* < 0.0001, Supplementary Fig. S4) confirming the trend effect observed with the multilevel logistic analysis (OR 0.48; 95% CI 0.38–0.61, *p* < 0.0001, Table 2). On the contrary, there was no statistically significant difference according to BMI (Table 2).

### Frequency of IgG positivity and correlation with age

**Figure 2:**
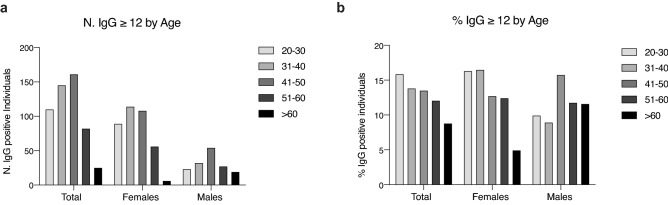
Frequency of IgG positivity (IgG ≥ 12 AU/mL) by age. (**a**,**b**) Histograms show the number (**a**) and the percentage (**b**) of positive individuals (IgG ≥ 12 AU/mL) divided by age range and sex on the whole population regardless of site (20–30: M, n = 22; F = 88; 31–40: M, n = 31; F, n = 114; 41–50: M, n = 53; F, n = 108; 51–60: M, n = 26; F, n = 56; > 60: M, n = 19; F, n = 6); in b, *p*-values were determined using Cuzick’s test for trend. *p* = 0.0374 (total); *p* = 0.0008 (female); *p* = 0.1498 (male); *p* = 0.0024 (interaction).

We then evaluated whether there was a difference in the positivity to IgG according to age. We found that there was a higher number of 31–50 years old IgG positive (≥ 12 AU/mL) individuals (Fig. 2a), but then when analyzing the frequency of positivity at the different age ranges we observed an age dependent reduction of IgG positive individuals (Fig. 2b). However, this age-dependency was primarily due to the female rather than the male population, particularly for subjects either young (20–40 yo) or older than 60 yo (test for heterogeneity of trend effect age between male and female *p* = 0.0024 Fig. 2b). In older than 60 yo, IgG positivity dropped from 12% in males to 5% in females (Table 2 and Fig. 2b).

### Frequency of IgG positivity and correlation with viral positivity at time of testing

Following the guidelines of the national health system, 516 out of 523 subjects with IgG ≥ 12 AU/ml underwent a nasopharyngeal swab for SARS-CoV-2 RNA viral detection. We found that 39 (7.6%) individuals resulted positive for viral RNA detection (Table 3). However, in 31 of these (79.5%), the subjects tested negative for at least one of the genomic sequences of the three SARS-CoV-2 gene targets: *E, RdRp* and *N*. To rule out that the negative population (3.8 < IgG < 12 AU/mL) comprised individuals in the early phases of viral infection, a sample of 283 (100%) individuals underwent a nasopharyngeal swab for SARS-CoV-2 RNA viral detection. All of them resulted negative to the swab suggesting that individuals with IgG < 12 AU/mL cannot be considered as under active viral infection (Table 3).

**Table 3:** Nasopharyngeal swab positivity in relation to IgG positivity.

	Total		Nasopharyngeal swab results											
			Negative			Positive low load			Positive			Positive low load + Positive		
	N	% of total	N	% of level	% of total	N	% of level	% of total	N	% of level	% of total	N	% of level	% of total
**IgG results**														
Negative (3.8 < IgG < 12AU/mL)	283	35.4	283	100.0	37.2									
Positive (≥ 12AU/mL)	516	64.6	477	92.4	62.8	31	6.0	100.0	8	1.6	100.0	39	7.6	100.0
Total	799	100.0	760	95.1	100.0	31	3.9	100.0	8	1.0	100.0	39	4.9	100.0

### Frequency of IgG positivity and correlation with professional status

We then evaluated the proportion of IgG positive individuals (≥ 12 AU/mL) across the different professional workers and found that there was no statistically significant difference when taking into account age, gender and their interaction (i.e. gender depending on age), smoking habits, BMI and location (Table 2) even though some professionals worked from home from the beginning of lockdown (such as the administrative staff), suggesting that their exposure to the virus was not occurring at work. We thus assessed which could have been the major risk of transmission of the virus on the basis of the self-reported questionnaire. Compared with group referring no interactions with COVID-19 positive people, the Odds ratio for IgG positivity was different among drivers (test for heterogeneity: *p* = 0.00001) ranging from 1.59 (95% CI 1.11–2.27), for interaction with colleagues to 4.73 (95% CI 2.93–7.65), for interactions with family members positive for COVID-19 (Tables 4, 5).

**Table 4:** Association of interactions with COVID-19 positive people and IgG positivity.

	Total		Negative (< 12AU/mL)		Positive (≥ 12AU/mL)	
	N	% of total	N	%	N	%
**Contagion modality**						
None	1073	27.0	1010	94.1	63	5.9
Colleague	853	21.5	740	86.8	113	13.2
Patient	1599	40.2	1346	84.2	253	15.8
Family member	138	3.5	95	68.8	43	31.2
Other	310	7.8	262	84.5	48	15.5
Total	3973	100.0	3453	86.9	520	13.1

**Table 5:** Summary measures of association of interactions with IgG positivity.

Contagion modality	Odds ratio	95% CI	
None	Ref		
Colleague	1.59	1.11	2.27
Patient	1.83	1.29	2.60
Family member	4.73	2.93	7.65
Other	2.05	1.32	3.18
P value (Wald test)	< 0.0001		

Multilevel logistic analysis, considering subjects nested in the hospital site. Adjusted for role, age (cut off = 60 years) gender, BMI, smoking habits.

### Frequency of IgG positivity and correlation with symptoms, comorbidities and vaccinations

**Figure 3:**
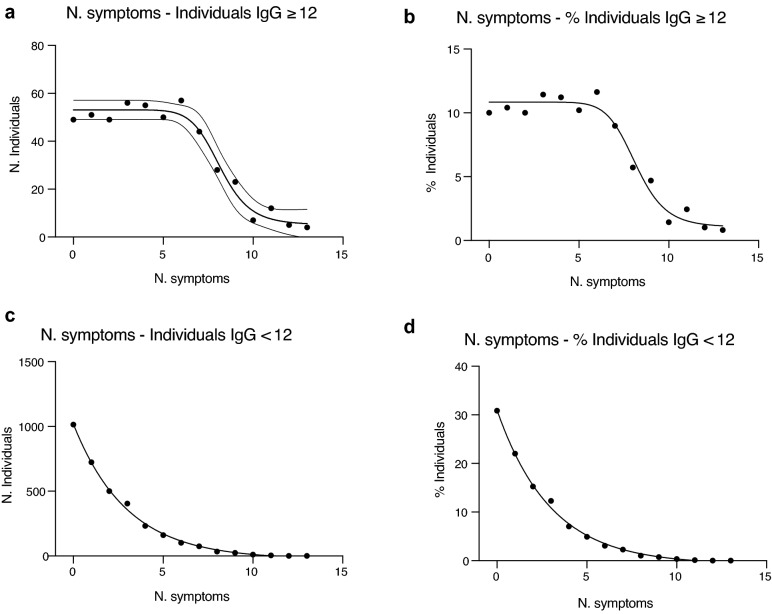
Distribution of the IgG ≥ 12 and IgG < 12 populations versus the number of symptoms. (**a**,**b**) Distribution of the IgG positive individuals (IgG ≥ 12 AU/mL) as number of individuals (**a**) or percentage of the population (**b**) versus the number of self-reported symptoms. The curve that best interpolated the data was a sigmoidal, four parameter logistic curve whereby X is the number of symptoms (R^2^ = 0.97). (**c**, **d**) Distribution of the IgG negative individuals (IgG < 12 AU/mL) as number of individuals (**c**) or percentage of the population (**d**) versus the number of self-reported symptoms. The curve that best interpolated the data was exponential (R^2^ = 0.9975).

We then evaluated whether there was a correlation between the number and typology of self-reported symptoms and the frequency of antibody response both as number of individuals (Fig. 3a,c) and as percentage on the whole population (Fig. 3b,d). As shown in Fig. 3, when analyzing individuals deemed to be positive on the basis of the amount of IgG (≥ 12 AU/mL) we observed a two phases decay: first a similar frequency (b) or number (a) of subjects with 0 to 7 concomitant symptoms, and then a drop of individuals at higher number of concomitant symptoms. This distribution followed a sigmoidal, four parameter logistic curve whereby X is the number of symptoms (R^2^ = 0.97). By contrast, in the population with IgG < 12 AU/mL we found a higher number (c) or frequency (d) of individuals with 0, 1 or 2 symptoms and the distribution was following an exponential curve (R^2^ = 0.9975). The multilevel logistic analysis showed that, besides pneumonia, among the symptoms, fever, anosmia/dysgeusia (loss of smell or taste) and chest pain were those that best characterized the IgG positive population, particularly when collated (Table 6).

**Table 6:** Multilevel Logistic model for symptoms adjusted for professional status, age, gender and their interaction, smoking habits, BMI.

Comparison	Odds ratio estimates	Lower 95% CI	Upper 95% CI	p-value
Fever	5.79	3.58	9.36	< 0.0001
Low-grade fever	1.41	0.86	2.33	0.1735
Cough	1.08	0.69	1.71	0.7266
Sore throath/runny nose	0.93	0.61	1.42	0.7332
Muscle pain	1.31	0.83	2.06	0.2453
Asthenia	0.92	0.57	1.47	0.7263
Anosmia/dysgeusia	11.51	7.06	18.77	< 0.0001
Gastrointestinal disorders	0.77	0.49	1.18	0.2299
Conjunctivitis	1.35	0.77	2.36	0.2887
Dyspnea	1.14	0.64	2.06	0.6539
Chest pain	2.17	1.26	3.74	0.0055
Tachycardia	0.64	0.38	1.09	0.0999
Pneumonia	9.00	1.34	60.66	0.0240

Indeed, dividing the population according to the number of symptoms, IgG positivity increased from 11.9% in the absence of symptoms to 42.8% in the presence of 5 symptoms or more (Table 7), and the value of the AUC derived by the multivariable model was 79%, while the combination of just fever versus anosmia/dysgeusia had an AUC of 78% (Supplementary Fig. S5). Fever with anosmia/dysgeusia, was present in 165 subjects (4.1%) of which 134 (81.2%) were associated with positive serology. Although sensitivity was 25.6% (134 with these symptoms /523 positive subjects), specificity was 99.1.% (3431 without these symptoms / 3820 negative subjects), yielding a positive likelihood ratio of 28.

**Table 7:** Association of symptoms and clinical manifestations with IgG positivity.

	Total		Negative (< 12AU/mL)			Positive (≥ 12AU/mL)		
	N	% of total	N	% of level	% of total	N	% of level	% of total
**Fever**								
No	3488	87.5	3179	91.1	91.8	309	8.9	59.1
Yes	497	12.5	283	56.9	8.2	214	43.1	40.9
**Low-grade fever**								
No	3557	89.3	3149	88.5	91.0	408	11.5	78.0
Yes	428	10.7	313	73.1	9.0	115	26.9	22.0
**Cough**								
No	3074	77.1	2750	89.5	79.4	324	10.5	62.0
Yes	911	22.9	712	78.2	20.6	199	21.8	38.0
**Sore throat/runny nose**								
No	2687	67.4	2391	89.0	69.1	296	11.0	56.6
Yes	1298	32.6	1071	82.5	30.9	227	17.5	43.4
**Muscle pain**								
No	2957	74.2	2707	91.5	78.2	250	8.5	47.8
Yes	1028	25.8	755	73.4	21.8	273	26.6	52.2
**Asthenia**								
No	3237	81.2	2948	91.1	85.2	289	8.9	55.3
Yes	748	18.8	514	68.7	14.8	234	31.3	44.7
**Anosmia/dysgeusia**								
No	3617	90.8	3348	92.6	96.7	269	7.4	51.4
Yes	368	9.2	114	31.0	3.3	254	69.0	48.6
**Gastrointestinal symptoms**								
No								
Yes	820	20.6	650	79.3	18.8	170	20.7	32.5
**Conjunctivitis**								
No	3583	89.9	3145	87.8	90.8	438	12.2	83.7
Yes	402	10.1	317	78.9	9.2	85	21.1	16.3
**Dyspnea**								
No	3726	93.5	3291	88.3	95.1	435	11.7	83.2
Yes	259	6.5	171	66.0	4.9	88	34.0	16.8
**Chest pain**								
No	3664	91.9	3235	88.3	93.4	429	11.7	82.0
Yes	321	8.1	227	70.7	6.6	94	29.3	18.0
**Tachycardia**								
No	3553	89.2	3112	87.6	89.9	441	12.4	84.3
Yes	432	10.8	350	81.0	10.1	82	19.0	15.7
**Pneumonia**								
No	3951	99.1	3457	87.5	99.9	494	12.5	94.5
Yes	34	0.9	5	14.7	0.1	29	85.3	5.5
**Other symptoms**								
No								
Yes	157	3.9	121	77.1	3.5	36	22.9	6.9
**Number of symptoms and clinical manifestations**								
0 symptoms	1380	34.6	1318	95.5	38.1	62	4.5	11.9
1 symptoms	814	20.4	758	93.1	21.9	56	6.9	10.7
2 symptoms	582	14.6	517	88.8	14.9	65	11.2	12.4
3 symptoms	411	10.3	350	85.2	10.1	61	14.8	11.7
4 symptoms	256	6.4	201	78.5	5.8	55	21.5	10.5
5 or more symptoms	542	13.6	318	58.7	9.2	224	41.3	42.8
**Fever and anosmia/dysgeusia**								
No	3285	82.4	3096	94.2	89.4	189	5.8	36.1
Anosmia/dysgeusia	203	5.1	83	40.9	2.4	120	59.1	22.9
Fever	332	8.3	252	75.9	7.3	80	24.1	15.3
Fever and anosmia/dysgeusia	165	4.1	31	18.8	0.9	134	81.2	25.6
Total	3985	100.0	3462	86.9	100.0	523	13.1	100.0

These results indicate that there are symptoms that best characterize the paucisymptomatic COVID-19 population and that when individuals present with fever and anosmia/dysgeusia they are likely to have SARS-CoV-2 infection.

When we analyzed if there was any correlation between IgG positivity and comorbidities or vaccination, we could not detect any and the odds ratios were all not statistically significant, however, the number of individuals with more than one comorbidity was low (8.5% of IgG positive individuals) making it difficult to draw major conclusions (Supplementary Tables S4a,b, S5a,b).

### IgG plasma levels and population characteristics

**Figure 4:**
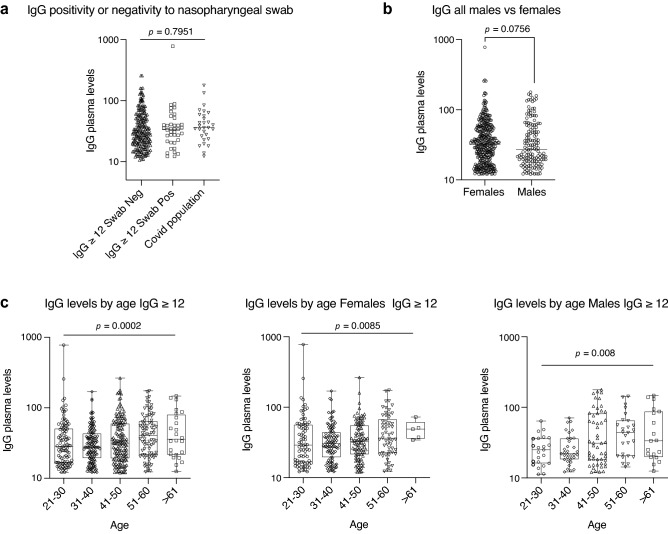
Distribution of IgG plasma levels in the different populations. (**a**) Distribution of IgG plasma levels in the cohort of IgG positive individuals (IgG ≥ 12 AU/mL) negative or positive for the nasopharyngeal swab or ascertained COVID-19 disease. (**b**) Distribution of IgG plasma levels in IgG positive individuals (IgG ≥ 12 AU/mL) divided by sex. (**c**) Distribution of IgG plasma levels in IgG positive individuals (IgG ≥ 12 AU/mL) divided by age ranges and sex. In (**a**) and (**b**) *p*-values were determined using Cochran Mantel-Haenzel test for trend. In (**c**) Cuzick’s test for trend was used: *p* = 0.0002 (total); *p* = 0.0085 (female); *p* = 0.008 (male); *p* = 0.4192 (interaction).

The frequency of IgG positive (≥ 12 AU/mL) individuals clearly reflected the increased exposure to the virus across the analyzed geographical areas, correlated with COVID-19-related symptoms and showed a higher proportion of positivity within young females and non-smoker subjects. However, an advantage of using a quantitative assay for IgG testing is that it is also possible to assess the magnitude of the anti-spike antibody response. Hence, we analyzed the data also in relation to IgG plasma levels. As we took into account a possible temporal confounding factor of symptoms detection and serological test, we compared the IgG plasma levels of swab positive or swab negative individuals with that of the ascertained COVID-19 population (between the months of March and April). We could not detect any statistically significant difference among IgG ≥ 12 subjects with nasopharyngeal swab positive or negative and the COVID-19 populations, suggesting that the positivity to the swab does not correlate with a higher IgG plasma level (Fig. 4a). However, we observed that physicians (including anesthesiologists and physiotherapists) had a lower level of plasmatic IgG as confirmed by statistically different odds ratios (Supplementary Table S6, *p* = 0.02 and odds ratios in Supplementary Fig. S6) this may reflect the use of PPE which might have reduced the viral load and hence also the development of an IgG immune response.

### IgG plasma levels BMI, gender and smoking

As we observed a higher proportion of positivity (IgG ≥ 12 AU/mL) within females that may indicate either a higher exposure to the virus, a higher incidence of infection or a higher ability to mount an immune response, we evaluated whether this difference was paralleled by increased IgG plasma levels. We found out that there was no difference between IgG plasma levels of males versus females, suggesting a similar magnitude of the anti-spike antibody response (Fig. 4b, Supplementary Table S6). However, when assessing a difference of IgG plasma levels across age ranges, there was a trend towards an increased level of plasma IgG towards the older age (*p* = 0.0002, total; *p* = 0.0085, females; *p* = 0.008, males) (Fig. 4c). Interestingly, while smoking seemed to inversely correlate with the frequency of IgG positive individuals, it did not have any effect on IgG plasma levels (Supplementary Fig. S7a) even in relation to the number of smoked cigarettes per day (Supplementary Fig. S7b and Supplementary Table S6). On the contrary, BMI, which was not influencing the frequency of IgG positive individuals, was affecting the plasma concentration of IgG: the higher the BMI, the higher the level of IgG (Supplementary Table S6, *p* = 0.0009).

### IgG plasma levels and symptoms

**Figure 5:**
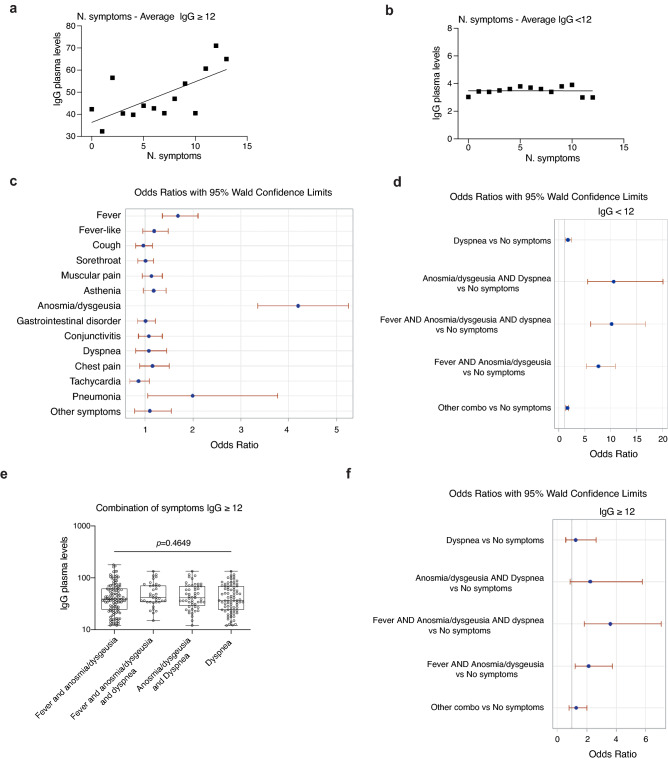
Distribution of IgG plasma levels in relation to symptoms. (**a**) Distribution of IgG plasma levels versus the number of self-reported symptoms in IgG positive individuals (IgG ≥ 12 AU/mL). (**b**) Distribution of IgG plasma levels versus the number of self-reported symptoms in IgG negative individuals (IgG < 12 AU/mL). (**c**) Association between symptoms and IgG plasma levels. *p* < 0.0001, LR test for global null hypothesis. Odds ratio (OR) calculated with logistic regression for ordinal data. (**d**) Analysis of plasma levels of the whole population in relation to a combination of symptoms, *p* < 0.0001. Odds ratio (OR) calculated with logistic analysis for ordinal data. (**e**) Distribution of IgG plasma levels versus selected symptoms (alone or in combination) in individuals with IgG ≥ 12 AU/mL. *p*-values evaluated using Kruskal–Wallis test with Dunn’s post-hoc test. (**f**) Analysis of plasma levels of positive subjects (IgG ≥ 12) in relation to a combination of symptoms, *p* = 0.0004. Odds ratio (OR) calculated with logistic analysis for ordinal data.

As we have shown that the distribution of the IgG positive (≥ 12 AU/mL) population followed a sigmoidal curve with a constant level of individuals up to 7 concomitant symptoms, we thus evaluated whether there was also a correlation between the plasma level of IgG and the number of symptoms. The distribution of IgG levels in the population versus the cumulative symptoms was very similar when analyzing the whole population or those of ICH and Gavazzeni which had different proportions of IgG positive individuals (Supplementary Fig. S8). They were characterized by similar areas under the curve (All: 571; ICH: 550; Gavazzeni: 522) and the respective Receiver Operating Characteristics (ROC) curves were perfect (100%), confirming maximal specificity and sensitivity of the IgG test at IgG ≥ 12 AU/mL (Supplementary Fig. S8). Interestingly, we observed a direct correlation between the number of concomitant symptoms and an increase in the level of plasma IgG (Fig. 5a, Supplementary Table S7; CI are reported in the table for the different symptoms and combinations thereof, *p* < 0.014). By contrast, the level of IgG in the population with values < 12 AU/mL was constant, regardless of the number of symptoms (Fig. 5b). When we analyzed the levels of IgG in relation to symptoms, we found that those subjects reporting fever, anosmia/dysgeusia, or pneumonia had all significant odds ratios (Fig. 5c). In addition, subjects with fever, cough, muscular pain, asthenia, anosmia/dysgeusia, dyspnea, chest pain, tachycardia or pneumonia, they all had higher IgG levels than those without symptoms (Supplementary Table S7, *p* values and CI are reported in the table for the different symptoms). This confirms that presence of symptoms correlates with higher IgG levels as shown in Fig. 5a. Further, combination of fever with anosmia/dysgeusia and/or dyspnea characterized populations with higher IgG plasma levels (Supplementary Table S7, *p* < 0.0001, CI are reported in the table for the different symptoms and combinations thereof), also when considering the whole population regardless of IgG positivity (Fig. 5d, *p* < 0.0001 in a LR test for global null hypothesis ordinal logistic analysis). When considering the level of IgG ≥ 12 the distribution of IgG plasma levels was very similar in subjects with these symptoms (Fig. 5e), however we found statistically significant differences in odds ratios when combining fever with anosmia/dysgeusia and fever with anosmia/dysgeusia and dyspnea (Fig. 5f, *p *= 0.0004 in a LR test for global null hypothesis ordinal logistic analysis). No statistically significant differences in IgG plasma levels were observed in relation to comorbidities. In steatosis/cirrhosis we had only two cases in which we found an inverse correlation but this should be confirmed on a higher number of individuals (*p* = 0.0211, CI 12.30–12.70, Supplementary Table S8). Moreover, we observed an interesting inverse correlation between pneumococcal vaccination and IgG plasma levels (*p* = 0.03, CI 15–29.40, Supplementary Table S9).

## Discussion

Here we report a comprehensive analysis of nearly 4000 individuals from different healthcare facilities representative of dramatically different levels of SARS-CoV-2 exposure in Lombardy, the most affected region by COVID-19 in Italy. We observed a range of positivity which strongly correlated with the geographical area of viral exposure from 3% in the Varese area to 43% in the Bergamo area, which were respectively the least and most COVID-19-affected Lombardy provinces. The proportion of IgG positive females was higher than that of males in all of the analyzed sites. Although differences in exposure cannot be completely accounted for, the effect of gender remained even after adjustment for site of work and other factors associated with an increased risk of susceptibility. This is in contrast with a recent meta-analysis showing that in USA and Europe, but not in East Asia, the prevalence of IgG antibodies was moderately higher among the male health care workers^13^. However, we found a lower proportion of IgG positive individuals in females older than 60 years old in age-matched males, suggesting that females are more likely to be infected—or to induce an IgG response—when young, and less likely at ages higher than 60 yo. This is quite intriguing as males and females are equally affected by COVID-19, but males have a worse prognosis^14^, suggesting that seroprevalence cannot provide any correlation with the clinical outcome. Through the use of a quantitative antibody test of IgG we were also able to assess the magnitude of the anti-spike antibody response. We found that younger males (below 40 yo) displayed reduced IgG plasma levels than older males (from 41 yo onwards). This is in line with a recent report in COVID-19 patients showing that younger patients developed lower titers of IgG^15^ and another one showing that higher IgG titers in the plasma of convalescent individuals were associated to older age and male sex^16^. Thus, also in the healthy population, younger males exposed to the virus develop a reduced antibody response. Moreover, the difference of IgG response between females and males in relation to age remains quite intriguing and we still have to understand its relevance with the higher incidence of COVID-19 in males^17^.Hence, it is very important when analyzing the serology to SARS-CoV-2 to take into account both age and sex.

Our study differs from the one reported by Sood and colleagues in Los Angeles County^18^ as we used a quantitative antibody test and analyzed a large hospital population which ranged from healthcare professionals, researchers and administrative staff from 7 different facilities. Indirectly, we show that it is rather the environment than the hospital professional exposure which dictates the probability of contracting SARS-CoV-2 infection. Indeed, we show a higher percentage of IgG positive individuals in areas with a higher incidence of COVID-19 and among subjects who had been in contact with COVID-19 affected relatives. Further, we observed a similar proportion of IgG positive individuals among healthcare professionals functioning at hospital and administrative staff working from home. We were also able to pinpoint 11.9% of IgG positive individuals which were completely asymptomatic and another 23.1% of paucisymptomatic subjects with 1 or 2 symptoms. This percentage differs from that observed in two recent studies analyzing SARS-CoV-2 infection via viral testing across different population types^19,20^. Among healthcare workers, 57% of the subjects were asymptomatic/paucisymptomatic^19^ while in a wider population in the Italian municipality of Vo’ Euganeo, that underwent lockdown soon after detecting the first case of COVID-19, the percentage of asymptomatics was 42.5^20^. This suggests that there is still a good proportion of individuals that probably do not develop antibodies, or develop them at low/undetectable levels. This would be in line with recent studies showing that IgG positivity is short-lived^21^ and relatives of COVID-19 patients carry T cells specific for SARS-CoV-2 but do not seroconvert, suggesting that also IgG positivity may not detect all individuals exposed to the virus^22^. However, if we expand our analysis to healthcare workers without symptoms but with IgG > 3.8 AU/mL, and not simply those IgG ≥ 12 AU/mL, which are considered ‘positive’ by the manufacturer of the test, the percentage of positivity raises to 40%. This may indicate that there may be different exposures to the virus which result in a wide range of levels of antibody production, including those between 3.8 and 12 AU/mL, but this could be a speculation. As in 283 subjects with IgG 3.8–12 AU/mL we performed a viral test, and it was negative, this indicates that they probably were exposed to the virus earlier and succeeded in eliminating it without a strong antibody response. Thus, it would be interesting to evaluate which serology test was used and which cut-off value was considered for determining IgG positivity in studies where IgG responses were negative, but T cell responses were present^22^. In addition, some of SARS-CoV-2 viral peptides activated T cells also from unexposed individuals suggesting that some cross-reactivity could occur, maybe in response to previous infections with common cold coronaviruses^23^.

Among the symptoms, those that characterized most the IgG positive population were fever and anosmia/dysgeusia. Indeed, 81.2% of individuals presenting both anosmia/dysgeusia and fever resulted SARS-CoV-2 infected, indicating that these symptoms are strongly associated to COVID-19.

Selected vaccines such as BCG have been suggested to increase pathogen-agnostic off-target resistance to infectious agents^24^. However, a recent report showed no differences in incidence of COVID-19 in BCG vaccinated versus non vaccinated patient population^25^. In line with this, we did not observe a correlation between IgG positivity and BCG vaccination.

Intriguingly, we observed an inverse correlation between induction of an antibody response and smoking habit. This may indicate either a lower incidence of SARS-CoV-2 infection in smokers, or their inability to induce an immune response to the virus. The former is more likely as a large study (114,545 individuals) in Israel recently showed that the risk of SARS-CoV-2 positive testing in smoking individuals is reduced by half^26^. Similarly, Norden et al. reported an inverse association between national smoking rates and COVID-19 mortality^27^ and this was confirmed in a meta-analysis showing an unexpectedly low prevalence of current smoking among patients with COVID-19 in China^28^. When analyzing the concentrations of IgG in plasma, we found no differences between males and females, nor in smoking habits. However, we observed that the presence of some symptoms such as fever, cough, muscular pain, asthenia, anosmia/dysgeusia and clinical manifestation such as pneumonia correlated with higher levels of IgG than in subjects without these symptoms. In addition, we found that the presence of comorbidities did not affect IgG plasma levels except for cirrhosis, but the frequency of individuals with multiple comorbidities was low (8.5% of IgG positive individuals with > 1 comorbidities). Subjects with cirrhosis had an inverse correlation with IgGs but the number of subjects was very low (2 out of 522) and this finding needs to be confirmed. The significance of this is unknown, but the finding that cirrhotic patients have higher risk of severe COVID^29^ may suggest that this condition may affect the correct development of antibodies. Finally, we found that although very few subjects had anti-pneumoccocal vaccination, these had a reduced level of IgG, while there was no difference among those vaccinated with influenza vaccine. A recent study has shown an inverse correlation between nasopharyngeal swab positivity and flu and pneumococcus vaccinations suggesting protection in vaccinated individuals^30^. Another report on the Italian population, based on regional and not on individual data, has shown an inverse correlation between COVID-19 cases and coverage of flu vaccinations, suggesting again a protective role of flu vaccination^31^. It is possible that flu vaccination, by inhibiting concomitant infection, may result in a better prognosis of the SARS-CoV-2 infected individuals which becomes evident only when analyzing COVID-19 cases and not asymptomatic or paucisymptomatic individuals. Why individuals vaccinated against pneumococcus should produce less antibodies remains to be evaluated. We found that transmission seemed to occur primarily when in contact with infected family members. This may be due to the lack of use of PPE in the household. Indeed, it has to be considered that the use of PPE may have impacted on transmission as in the hospital it was implemented starting from February 1st 2020, consistently we observed a reduction of IgG plasma levels in physicians. This finding is in line with a recent report on testing the serology of healthcare workers and their families^32^.

In conclusion, our study has some limitations, as it is monitoring healthcare workers, and thus it does not reflect a comprehensive population as a good proportion of professionals are women and there is an underrepresentation of the elderly due to working age limit. However, we show that antibody testing can identify the individuals that were exposed to SARS-CoV-2 and is a powerful tool to retrospectively evaluate viral diffusion, even in asymptomatic individuals. Another limitation of the study is that we may have missed the population of subjects which do not develop an antibody response or that is reduced over time. As our study is ongoing we will assess the evolution of the IgG response over a planned follow up of one year, allowing for measuring the duration of the antibody response. Should a second wave of SARS-CoV-2 infection occur, the wide range of IgG serology in the different sites will be particularly valuable as it will allow us to assess the role of antibodies in viral protection. The results presented here suggest that hospital health care professionals, researchers and administrative staff can provide invaluable information to assess variables affecting the immune response to SARS-CoV-2 as a snapshot and during the follow-up.

## Methods

### Clinical study

The study has been approved by the international review board of Istituto Clinico Humanitas for all participating institutes (clinicaltrial.gov NCT04387929). Accrual was on a voluntary basis: it started on April 28th 2020 and more than 65% of employees (n = 3985) participated as of May 16th 2020. An international medical school (Humanitas University, Pieve Emanuele (MI)) and 6 different hospitals participated to the study: Istituto Clinico Humanitas (ICH), Rozzano (MI); Humanitas Gavazzeni, Bergamo; Humanitas Castelli, Bergamo; Humanitas Mater Domini (HMD), Castellanza (VA); Humanitas Medical Care (HMC), Varese (VA); Humanitas San Pio X, Milano (MI). All participants signed an informed consent and filled a questionnaire (Supplementary Data S1) before blood collection. We analyzed COVID-19-associated symptoms and clinical manifestations as tachycardia and/or pneumonia (the latter confirmed by TC). We considered “asymptomatics” subjects without any symptoms; “paucisymptomatics” individuals that developed 1 or 2 symptoms; “symptomatics” individuals with more than 3 symptoms. Clinical manifestations (tachycardia and pneumonia) were always associated with at least other 2 symptoms. In order to be tested, subjects had to fill in the questionnaire. Only after filling the questionnaire in its entirety, would the individuals be scheduled for blood sampling. Hence, we obtained a 100% complete data. None of the participants were enrolled at the time of symptoms. Thus, when the serological test was performed, they were either asymptomatics or the symptoms had disappeared. If they resulted positive for IgG (IgG ≥ 12 AU/mL) they underwent a nasopharyngeal swab for SARS-CoV-2 viral RNA detection to exclude being actively infected. We analyzed the mean of serology date versus the appearance of COVID-19 symptoms and found that they were tested around 43 ± 17 days after symptoms.

### SARS-CoV-2 RT-PCR

Nasopharyngeal swab were tested with a commercial RT-PCR assay (Allplex 2019-nCoV Assay—Seegene, Seoul, South Korea), according to manufacturer’s instruction. RNA extraction was performed using Seegene Nimbus, a liquid handler workstation, Real-time PCR was run on a CFX96 TMDx thermocycler (Bio-Rad Laboratories, Inc, CA, USA) and subsequently interpreted by Seegene’s Viewer software. The test target three viral genes (*E, RdRp* and *N*).

### IgG determination

For the determination of IgG anti SARS-CoV-2 the Liaison SARS-CoV-2 S1/S2 IgG assay (DiaSorin, Saluggia (VC), Italy) was used^33^. The method is an indirect chemiluminescence immunoassay for the determination of anti-S1 and anti-S2 specific antibodies. Intra- and inter-assay coefficient of variation are < 1.9% and < 3.7% respectively. In the datasheet of the test, in a cohort of 304 COVID-19 patients 97.5% of the samples above 15 AU/mL and 86% of those with IgG > 12 AU/mL resulted positive for neutralizing antibodies with a titer > 1:40 in the Plaque Reduction Neutralization Test (PRNT).

The sensitivity of the test as reported by the manufacturer is 90.4% (79.4–95.8%) while the specificity is 98.5% (97.5–99.2%).

### Statistics

This was a cross sectional study aimed at determining the frequency of plasma anti- SARS-CoV-2 IgG antibodies in nearly 4000 (3985) employees of 7 different healthcare facilities located in Lombardy.

The serological determination was offered to all the employees of the involved sites and the anticipated refusal rate was assumed to be 10–15%. The overall IgG positivity was assumed to be around 10–15% (between 400 and 600 subjects).

Primary endpoint was the number of test positive subjects (523 out of 3985). Given the study size, the study was able to estimate the overall positivity with a width of 95% confidence interval equal to 2% and the positivity for subgroups of at least 200 patients with a width of 95% confidence interval equal to 9%. No power analysis was performed to calculate the sample size.

As secondary endpoints we aimed to investigate the factors associated to plasma anti-SARS-CoV-2 IgG positivity.

Basic demographic characteristics, including age, sex, smoking status, comorbidities, previous vaccinations and symptoms were recorded and their association with IgG positivity was tabulated in contingency tables and described by means of relative and absolute frequencies.

The association between these characteristics and IgG positivity was assessed by univariate logistic model and measured with the odds ratio (OR) and 95% Confidence Interval (CI) for each factor. A multivariable analysis, was also performed in order to assess the effect of characteristics of interest after adjustment for the other variables.

Furthermore, since our data have clustered structures as patients were clustered within hospitals, a multilevel model was also used to analyse data. The binary outcome (positive vs. negative serological assessment) was chosen and analysis was approached with a generalized linear model with the logic link, which is the logistic regression model. In our multilevel analysis there was a patient level and an hospital level.

We produced two models. In the first one we want to assess the effect of role, gender, age, BMI and cigarette smoking on the risk of positive serology. We estimated a random intercept model for hospital profiling and treat the hospital effects as random effects only. The role of subjects was considered related to the hospital attributes, while the subject level attributes were age (grouped in > 60, ≤ 60 years) and gender, BMI (≤ 20, ≤ 25, ≤ 30, ≤ 35, > 35) and number of smoked cigarettes/day (no, ≤ 10, ≤ 20 > 20). BMI and cigarette smoking were considered as continuous variables. The others variables are all categorical. The interaction term of age*gender was included in the model.

In the second one we explored the relationship between vaccinations, symptoms and serological positivity, after adjustment for the previous factors, still considering a hierarchical model. This was obtained adding the symptoms, defined as binary variables, to the previous models. Summary statistical measures were the odds ratio with relative confidence interval. SAS Glimmix procedure (SAS 9.4 release) was used for the purpose of analysis.

The same approach was used also for analysis of plasma levels. SAS Mixed procedure (SAS 9.4 release) was used for the purpose of analysis.

Kruskal–Wallis and non-parametric tests for trend (Cuzick’s and Mantel-Haenzel test, when appropriate) have been used for multiple comparisons using Prism 8 Graphpad. For plasma levels, odds ratio and relative 95%CI based on Wald Test presented in the figures were calculated using logistic model applied to ordinal data.

## Supplementary Information

Supplementary Information.

## Data Availability

Data supporting the findings of this study are available within the paper and its Supplementary Information files. The dataset generated during and/or analysed during the current study is available from the corresponding author on reasonable request.
