# Supplementary material for: SARS-CoV-2 serology in 4000 health care and administrative staff across seven sites in Lombardy, Italy
Source: Sci Rep. 2021 Jun 10;11:12312. doi: 10.1038/s41598-021-91773-4 (PMC8192543; doi:10.1038/s41598-021-91773-4)
Supplement: Supplementary file 1 — Supplementary Information. [file 41598_2021_91773_MOESM1_ESM.pdf]

Supplementary Information

SARS-CoV-2 serology in 4000 health care and administrative staff across seven sites in Lombardy, Italy

Maria Teresa Sandri[1¶], Elena Azzolini[1¶], Valter Torri[2], Sara Carloni[3], Chiara Pozzi[1], Michela Salvatici[1], Michele Tedeschi[1], Massimo Castoldi[5], Alberto Mantovani[1,3,4] and Maria Rescigno[1,3*]

¶These authors contributed equally

* Corresponding author

E-mail: maria.rescigno@hunimed.eu

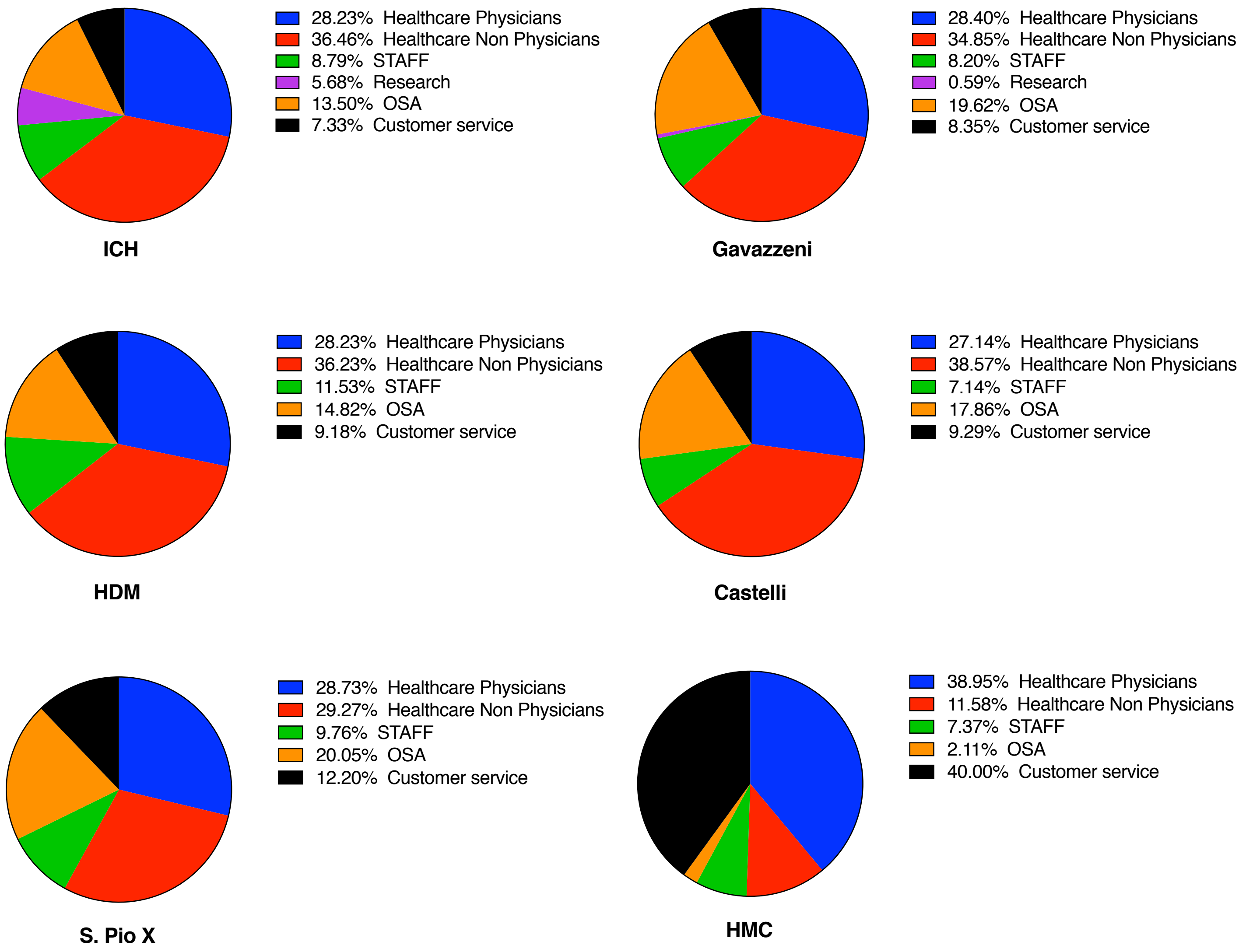

Supplementary Figure 1. Distribution of personnel in the six hospitals analyzed.
Pie charts show the percentage of healthcare workers participating to the study by site.
In blue healthcare physicians, in red healthcare non physicians, in green administrative staff (STAFF), in violet research personnel, in orange nurses (OSA), in black customer service (check-in, admissions).

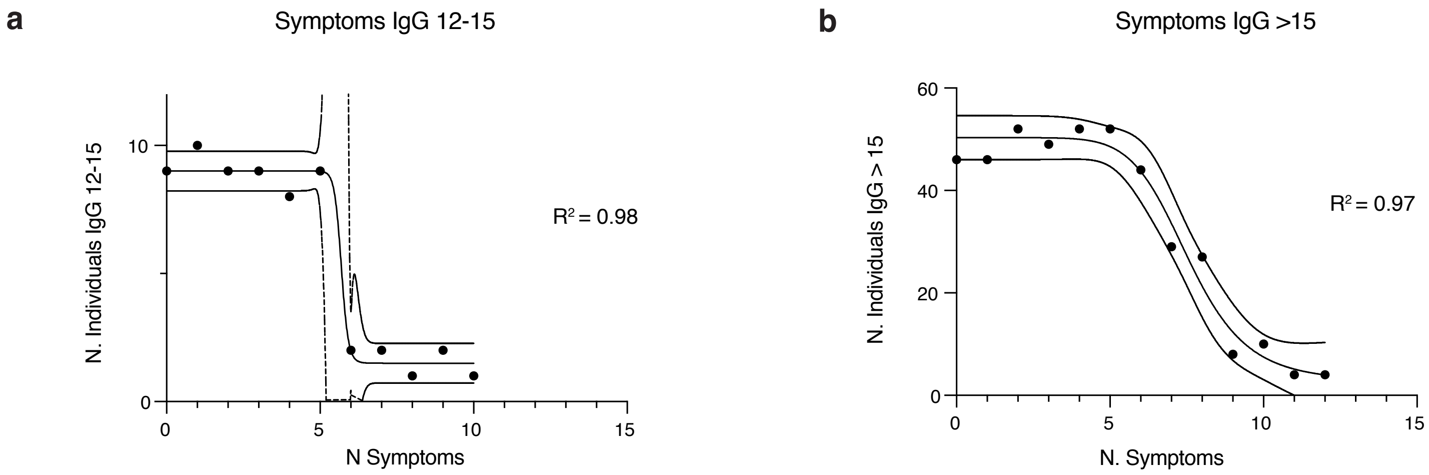

Supplementary Figure 2. Correlation between IgG positivity and number of symptoms.
a, **b**, Distribution of the IgG equivocal population (IgG 12-15 AU/mL) (**a**) and IgG truly positive population (IgG>15 AU/mL) (**b**) as number of individuals versus the number of symptoms. Both populations follow a sigmoidal, four parameter logistic curve whereby X is the number of symptoms. Distribution R^2 numbers are reported to demonstrate the fitness of the curve.

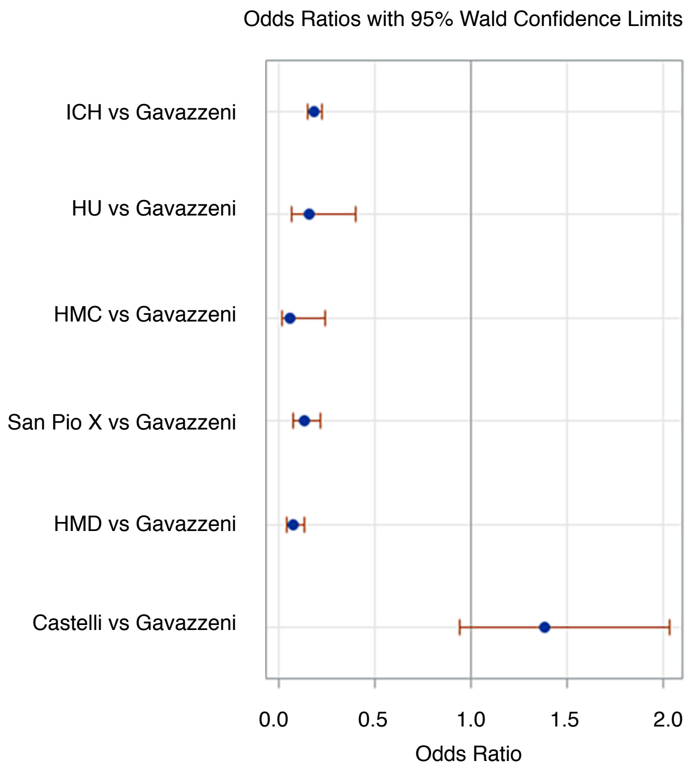

Supplementary Figure 3. Association between site and IgG positivity.
Odds-ratio calculated with multilevel logistic analysis.

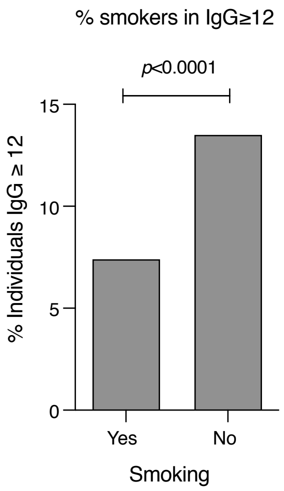

Supplementary Figure 4. Distribution of the IgG positive population (IgG≥12 AU/mL) according to smoke. Odds ratio calculated with multilevel logistic analysis **(**OR=0.45; 95%CI 0.34-0.60, p<0.0001).

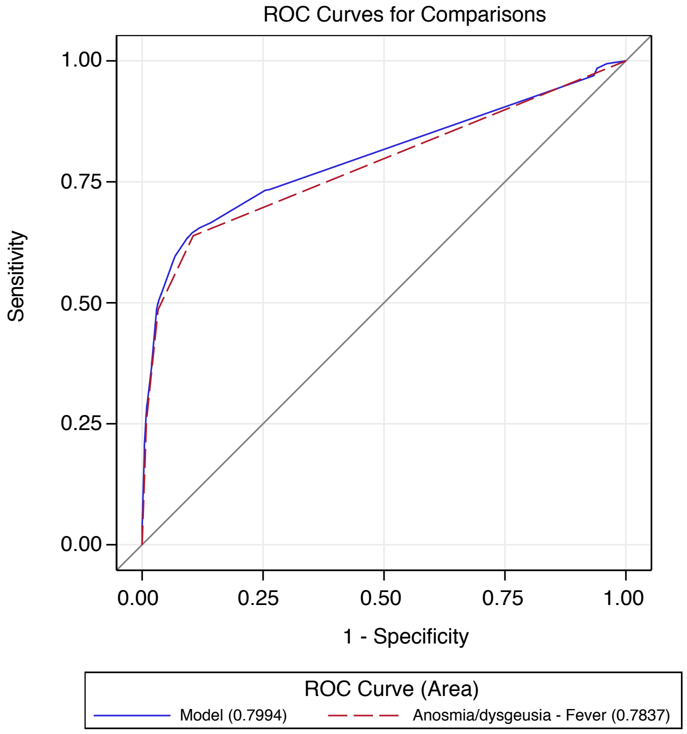

ROC Association Statistics				
ROC Model	Mann-Whitney			
	Area	Std Err	95% CI	
Model considering all symptoms	0.799	0.012	0.775	0.823
Model considering only fever + anosmia/dysgeusia	0.784	0.011	0.761	0.806

Supplementary Figure 5. ROC analysis of relationship between IgG positivity and symptoms. Logistic model.

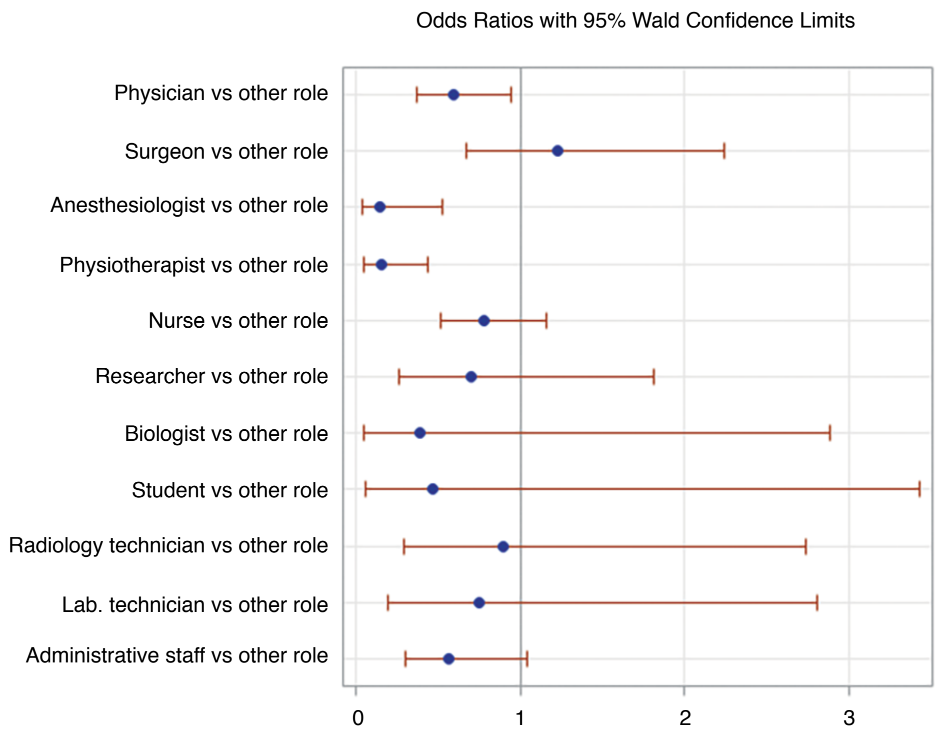

Supplementary Figure 6. Association between role and IgG plasma levels. Odds-ratio calculated with logistic regression applied to ordinal data.

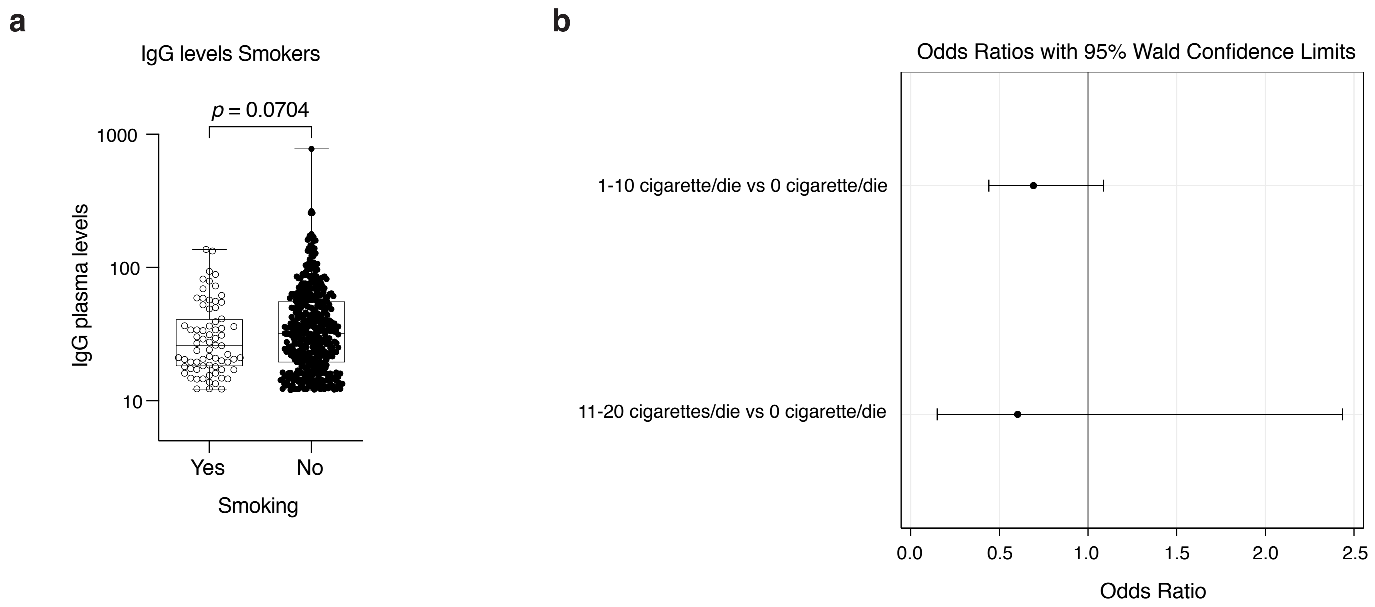

Supplementary Figure 7. Association between smoking and IgG plasma levels.
a, Distribution of the IgG positive population (IgG≥12 AU/mL) as plasma levels divided by smoking habit (yes or no). *p*-value was calculated using Kruskal-Wallis test; **b**, Odds-ratio calculated with logistic regression applied to ordinal data. LR test for global null hypothesis, p=0.207.

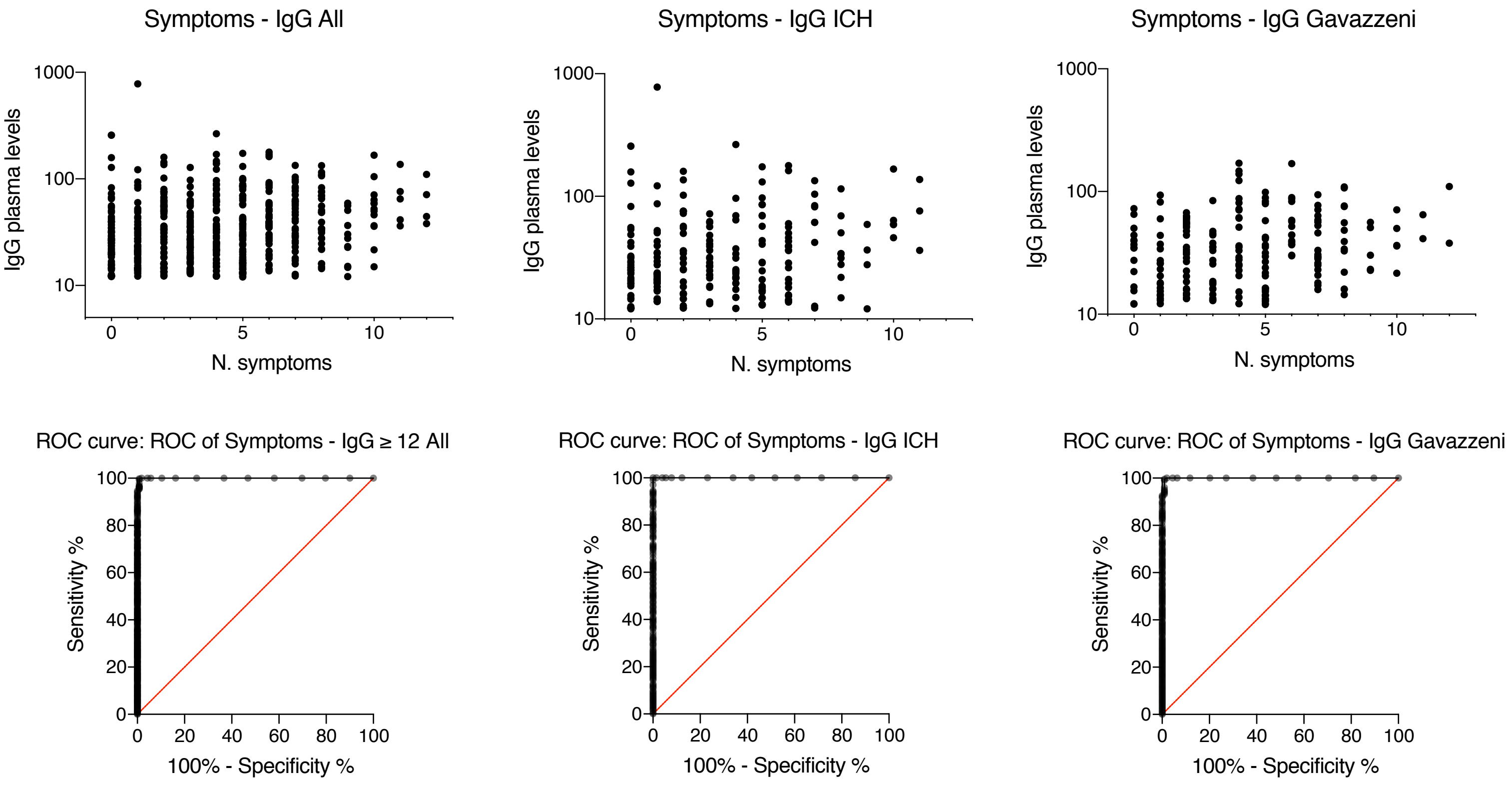

Supplementary Figure 8. IgG plasma level distribution in the positive population (IgG ≥ 12 AU/mL) versus symptoms across all and the two major sites, ICH and Gavazzeni. The areas under the curve are respectively: 571 All, 550 ICH, 522 Gavazzeni. Below each graph is reported the corresponding ROC curve. All of them show 100% of sensitivity and specificity.

Supplementary Table 1 Characteristics of the population in relation to IgG positivity

	Total		Negative (<12AU/mL)			Positive (≥12AU/mL)		
	N	% of total	N	% of level	% of total	N	% of level	% of total
**Gender Male**								
No	2660	66.8	2288	86.0	66.1	372	14.0	71.1
Yes	1325	33.2	1174	88.6	33.9	151	11.4	28.9
**Work site**								
Humanitas Rozzano (ICH)	2558	64.2	2329	91.0	67.3	229	9.0	43.8
Humanitas University (HU)	64	1.6	59	92.2	1.7	5	7.8	1.0
Humanitas Medical Care (HMC)	67	1.7	65	97.0	1.9	2	3.0	0.4
Humanitas San Pio X	250	6.3	234	93.6	6.8	16	6.4	3.1
Humanitas Mater Domini (HMD)	341	8.6	328	96.2	9.5	13	3.8	2.5
Humanitas Castelli	133	3.3	76	57.1	2.2	57	42.9	10.9
Humanitas Gavazzeni	572	14.4	371	64.9	10.7	201	35.1	38.4
**Profession**								
Physician	659	16.5	569	86.3	16.4	90	13.7	17.2
Surgeon	287	7.2	245	85.4	7.1	42	14.6	8.0
Anesthesiologist	112	2.8	104	92.9	3.0	8	7.1	1.5
Physiotherapist	72	1.8	60	83.3	1.7	12	16.7	2.3
Nurse	1014	25.4	859	84.7	24.8	155	15.3	29.6
Researcher	123	3.1	109	88.6	3.1	14	11.4	2.7
Biologist	50	1.3	47	94.0	1.4	3	6.0	0.6
Student	28	0.7	25	89.3	0.7	3	10.7	0.6
Radiology Technician	94	2.4	84	89.4	2.4	10	10.6	1.9
Lab. Technician	79	2.0	72	91.1	2.1	7	8.9	1.3
Staff	315	7.9	275	87.3	7.9	40	12.7	7.6
Other role	1152	28.9	1013	87.9	29.3	139	12.1	26.6
**Total**	3985	100.0	3462	86.9	100.0	523	13.1	100.0

Supplementary Table 2 Distribution of IgG positivity by sex across the different sites							
		Serology				Total	
		negative		positive			
		N	%	N	%	N	%
**Working site**							
Humanitas Rozzano (ICH)		2329	91.0	229	9.0	2558	100.0
Humanitas University (HU)		59	92.2	5	7.8	64	100.0
Humanitas Medical Care (HMC)		65	97.0	2	3.0	67	100.0
Humanitas San Pio X		234	93.6	16	6.4	250	100.0
Humanitas Mater Domini (HMD)		328	96.2	13	3.8	341	100.0
Humanitas Castelli		76	57.1	57	42.9	133	100.0
Humanitas Gavazzeni		371	64.9	201	35.1	572	100.0
Humanitas Milan		3015	91.9	265	8.1	3280	100.0
**Males**							
No		2288	86.0	372	14.0	2660	100.0
Yes		1174	88.6	151	11.4	1325	100.0
**Working site**	**Males**						
Humanitas Rozzano (ICH)	No	1515	90.7	156	9.3	1671	100.0
	Yes	814	91.8	73	8.2	887	100.0
Humanitas University (HU)	No	37	90.2	4	9.8	41	100.0
	Yes	22	95.7	1	4.3	23	100.0
Humanitas Medical Care (HMC)	No	48	96.0	2	4.0	50	100.0
	Yes	17	100.0	.	.	17	100.0
Humanitas San Pio X	No	152	93.3	11	6.7	163	100.0
	Yes	82	94.3	5	5.7	87	100.0
Humanitas Mater Domini (HMD)	No	230	96.2	9	3.8	239	100.0
	Yes	98	96.1	4	3.9	102	100.0
Humanitas Castelli	No	56	57.1	42	42.9	98	100.0
	Yes	20	57.1	15	42.9	35	100.0
Humanitas Gavazzeni	No	250	62.8	148	37.2	398	100.0
	Yes	121	69.5	53	30.5	174	100.0
Humanitas Milan	No	1982	91.6	182	8.4	2164	100.0
	Yes	1033	92.6	83	7.4	1116	100.0
**Total**		3462	86.9	523	13.1	3985	100.0

Supplementary Table 3 Sensitivity, specificity and positive Likelihood ratio of symptoms/clinical manifestations						
**Symptoms**	**True pos**	**False pos**	**True neg**	**False neg**	**LR pos**	**LR neg**
Fever	40.9%	8.2%	91.8%	59.1%	5.01	1.55
Low-grade Fever	78.0%	9.0%	91.0%	78.0%	8.63	1.17
Cough	38.0%	20.6%	79.4%	62.0%	1.85	1.28
Sore Throat/Runny nose	43.4%	30.9%	69.1%	56.6%	1.40	1.22
Muscle pain	52.2%	21.8%	78.2%	62.0%	2.39	1.26
Asthenia	44.7%	14.8%	85.2%	55.3%	3.01	1.54
Anosmia/Dysgeusia	48.6%	3.3%	96.7%	51.4%	14.75	1.88
Gastrointestinal symptoms	32.5%	18.8%	81.2%	67.5%	1.73	1.20
Conjunctivitis	16.3%	9.2%	90.8%	83.7%	1.77	1.08
Dyspnea	16.8%	4.9%	95.1%	83.2%	3.41	1.14
Chest pain	18.0%	6.6%	93.4%	82.0%	2.74	1.14
Tachycardia	15.7%	10.1%	89.9%	84.3%	1.55	1.07
Pneumonia	5.5%	0.1%	99.9%	94.5%	38.39	1.06
Other symptoms	6.9%	3.5%	96.5%	93.1%	1.97	1.04
Fever & Anosmia/Dysgeusia	25.6%	0.9%	99.1%	74.4%	28.61	1.33

Supplementary Table 4a Correlation between comorbidities and IgG positivity						
	**Total**		**Negative (<12AU/mL)**		**Positive (≥12AU/mL)**	
	N	% of total	N	%	N	%
**Chronic obstructive pulmonary disease (COPD)**						
No	3968	99.6	3448	86.9	520	13.1
Yes	17	0.4	14	82.4	3	17.6
**Asthma**						
No	3741	93.9	3249	86.8	492	13.2
Yes	244	6.1	213	87.3	31	12.7
**Dyslipidemia**						
No	3619	90.8	3138	86.7	481	13.3
Yes	366	9.2	324	88.5	42	11.5
**Active neoplasia (NPL)**						
No	3981	99.9	3458	86.9	523	13.1
Yes	4	0.1	4	100.0	.	.
**History of NPL**						
No	3874	97.2	3365	86.9	509	13.1
Yes	111	2.8	97	87.4	14	12.6
**Chronic heart failure**						
No	3982	99.9	3459	86.9	523	13.1
Yes	3	0.1	3	100.0	.	.
**Hypertension**						
No	3637	91.3	3159	86.9	478	13.1
Yes	348	8.7	303	87.1	45	12.9
**History of CHD**						
No	3960	99.4	3438	86.8	522	13.2
Yes	25	0.6	24	96.0	1	4.0
**Atrial Fibrillation**						
No	3957	99.3	3439	86.9	518	13.1
Yes	28	0.7	23	82.1	5	17.9
**History of TIA/stroke**						
No	3973	99.7	3451	86.9	522	13.1
Yes	12	0.3	11	91.7	1	8.3
**Steatosis/Cirrhosis**						
No	3968	99.6	3447	86.9	521	13.1
Yes	17	0.4	15	88.2	2	11.8
**Other Hepatic diseases**						
No	3962	99.4	3442	86.9	520	13.1

Supplementary Table 4a Correlation between comorbidities and IgG positivity

	Total		Negative (<12AU/mL)		Positive (≥12AU/mL)	
	N	% of total	N	%	N	%
Yes	23	0.6	20	87.0	3	13.0
**Chronic kidney failure**						
No	3977	99.8	3455	86.9	522	13.1
Yes	8	0.2	7	87.5	1	12.5
**Rheumatoid Arthritis**						
No	3921	98.4	3410	87.0	511	13.0
Yes	64	1.6	52	81.3	12	18.8
**Other Immune system diseases**						
No	3726	93.5	3242	87.0	484	13.0
Yes	259	6.5	220	84.9	39	15.1
**Diabetes mellitus**						
No	3943	98.9	3421	86.8	522	13.2
Yes	42	1.1	41	97.6	1	2.4
**Gout**						
No	3980	99.9	3457	86.9	523	13.1
Yes	5	0.1	5	100.0	.	.
**Other comorbidities**						
No	3584	89.9	3101	86.5	483	13.5
Yes	401	10.1	361	90.0	40	10.0
**Number of comorbidities**						
0 comorbidity	2538	63.7	2192	86.4	346	13.6
1 comorbidity	1062	26.6	929	87.5	133	12.5
2 comorbidities	273	6.9	244	89.4	29	10.6
3 comorbidities	82	2.1	71	86.6	11	13.4
4 or more comorbidities	30	0.8	26	86.7	4	13.3
**Total**	3985	100.0	3462	86.9	523	13.1

Supplementary Table 4b Summary measures of association of comorbidity with IgG positivity				
**Comorbidity**	**Odds ratio**	**95% CI**		***P* value**
COPD	3.17	0.79	12.71	0.1034
Asthma	0.91	0.59	1.41	0.6664
Dyslipidemia	0.94	0.64	1.39	0.7661
Active NPL	NM	NM	NM	NM
History of NPL	0.98	0.52	1.82	0.9398
Chronic heart failure	NM	NM	NM	NM
Hypertension	1.13	0.76	1.67	0.5401
History of CHD	0.52	0.06	4.30	0.5452
Atrial Fibrillation	1.29	0.39	4.26	0.6712
History of TIA/Stroke	0.97	0.11	8.41	0.9757
Steatosis/ Cyrrhosis	0.81	0.16	3.99	0.7972
Other hepatic diseases	0.47	0.10	2.24	0.3456
Chronic kidney failure	0.50	0.05	4.93	0.5493
Rheumatoid Arthritis	1.92	0.91	4.04	0.0858
Other Immune system diseases	1.08	0.72	1.64	0.7013
Diabetes mellitus	0.19	0.02	1.42	0.1055
Gout	NM	NM	NM	NM
Other comorbidities	0.65	0.44	0.95	0.0274
Number of comorbidities	0.92	0.80	1.06	0.2414

Multilevel logistic analysis, considering subjects nested in the hospital site. Adjusted for role, age (cut off = 60 years), gender, BMI, smoking habits. NM = not measurable

Supplementary Table 5a Correlation between IgG positivity and vaccinations						
	**Total**		**Negative (<12AU/mL)**		**Positive (≥12AU/mL)**	
	N	% of total	N	%	N	%
**Influenza vaccine 2019/2020**						
No	2774	69.6	2414	87.0	360	13.0
Yes	1211	30.4	1048	86.5	163	13.5
**Anti-pneumococcal vaccine**						
No	3851	96.6	3351	87.0	500	13.0
Yes	134	3.4	111	82.8	23	17.2
**Anti-BCG vaccine**						
No	3599	90.3	3122	86.7	477	13.3
Yes	386	9.7	340	88.1	46	11.9
**Other vaccines**						
No	3703	92.9	3214	86.8	489	13.2
Yes	282	7.1	248	87.9	34	12.1
**Number of vaccinations**						
0 vaccination	2303	57.8	2000	86.8	303	13.2
1 vaccination	1404	35.2	1224	87.2	180	12.8
2 vaccinations	231	5.8	197	85.3	34	14.7
3 or more vaccinations	47	1.2	41	87.2	6	12.8
**Total**	3985	100.0	3462	86.9	523	13.1

Supplementary Table 5b Summary measures of association of vaccinations with IgG positivity				
**Type of vaccination**	**Odds ratio**	**95% CI**		***P* value**
Influenza vaccine 2019/2020	1.15	0.91	1.44	0.2368
Anti-pneumococcal vaccine	1.38	0.81	2.34	0.2380
Anti-BCG Vaccine	0.82	0.57	1.19	0.3029
Other	0.71	0.46	1.09	0.1129
Multilevel logistic analysis, considering subjects nested in the hospital site. Adjusted for role, age (cut off = 60 years), gender, BMI, smoking habits.				

Supplementary Table 6 Medians and percentiles of IgG plasma levels in relation to age, BMI, smoking, site, professional status

	Positivity ≥12AU/mL						P value*
	N	Median	25°centile	75°centile	Min	Max	
**Age> 60 yo**							0.1212
No	497	30.80	18.50	53.80	12.10	778.00	
Yes	25	37.40	22.00	72.70	12.50	147.00	
**Males**							0.4793
No	371	32.00	19.20	53.90	12.10	778.00	
Yes	151	27.10	18.50	56.20	12.10	178.00	
**BMI**							0.0009
BMI lt 20	64	28.20	16.00	53.85	12.80	128.00	
BMI lt 25	252	28.70	17.80	47.95	12.10	170.00	
BMI lt 30	127	34.70	21.80	59.10	12.10	169.00	
BMI lt 35	34	46.20	19.70	82.10	12.20	265.00	
BMI ge 35	9	38.70	23.90	62.30	19.70	778.00	
**Number of smoked cigarettes**				.	.		0.0704
0 cigarette/die	451	31.90	19.40	56.10	12.10	778.00	
1-10 cigarette/die	65	25.90	18.00	41.20	12.20	137.00	
11-20 cigarette/die	6	23.30	18.00	34.90	15.50	61.70	
21 or more cigarette/die	0	.	.	.	.	.	
**Working site**							0.2922
Humanitas Rozzano (ICH)	229	28.70	18.60	52.60	12.10	778.00	
Humanitas University (HU)	5	27.60	25.10	67.30	14.20	82.70	
Humanitas Medical Care (HMC)	2	20.90	19.20	22.60	19.20	22.60	
Humanitas San Pio X	16	43.35	26.25	73.80	13.40	128.00	
Humanitas Mater Domini (HMD)	13	19.00	16.30	26.70	14.40	143.00	
Humanitas Castelli	57	33.60	21.30	50.30	12.50	133.00	
Humanitas Gavazzeni	200	33.30	18.00	55.10	12.20	170.00	
Humanitas Milan	265	28.50	18.50	55.30	12.10	778.00	
**Profession**							0.0200
Physician	89	28.60	15.40	60.40	12.20	170.00	
Surgeon	42	36.25	21.60	72.10	12.50	178.00	
Anesthesiologist	8	15.10	13.50	37.90	12.80	76.10	
Physioterapist	12	16.60	13.40	21.75	12.20	64.10	
Nurse	155	31.80	20.40	50.90	12.20	778.00	
Research	14	28.20	15.00	51.90	12.20	257.00	
Laboratory technician	3	34.30	12.10	40.60	12.10	40.60	
Student	3	23.20	14.20	67.30	14.20	67.30	
Lab. technician	17	31.90	23.80	48.70	16.20	63.10	
Staff	17	31.90	23.80	48.70	16.20	63.10	
Other	179	31.20	20.30	56.10	12.10	265.00	
**Total**	522	31.20	18.60	54.80	12.10	778.00	

Kruskal Wallis test statistic for categorical variable; Cuzick's test for trend for ordinal variables.

Supplementary Table 7 Medians and percentiles of IgG plasma levels in relation to symptoms/clinical manifestations

	Positivity ≥12AU/mL						P value*
	N	Median	25°centile	75°centile	Min	Max	
**Fever**							<0.0001
No	308	27.10	17.20	46.50	12.10	778.00	
Yes	214	36.40	22.60	61.70	12.10	265.00	
**Low-grade Fever**							0.0080
No	407	29.40	18.10	50.20	12.10	778.00	
Yes	115	37.70	22.00	63.10	12.20	170.00	
**Cough**							<0.0001
No	323	27.10	16.60	48.70	12.10	778.00	
Yes	199	37.70	22.80	61.30	12.10	178.00	
**Sore throat/Runny nose**							0.9538
No	296	31.40	18.00	57.15	12.10	778.00	
Yes	226	30.85	19.50	50.40	12.10	170.00	
**Muscle pain**							0.0223
No	250	27.50	18.30	48.90	12.10	257.00	
Yes	272	33.80	19.35	58.75	12.10	778.00	
**Asthenia**							0.0012
No	289	26.90	18.40	47.50	12.10	778.00	
Yes	233	36.40	20.80	60.50	12.10	178.00	
**Anosmia/Dysgeusia**							0.0119
No	269	27.50	17.60	50.50	12.10	778.00	
Yes	253	34.30	21.30	57.20	12.10	265.00	
**Gastrointestinal symptoms**							0.7627
No	352	31.20	19.50	52.30	12.10	778.00	
Yes	170	31.50	17.70	57.10	12.10	174.00	
**Conjunctivitis**							0.3557
No	438	30.20	18.40	55.30	12.10	778.00	
Yes	84	35.60	22.40	52.20	12.20	131.00	
**Dyspnea**							0.0044
No	434	29.25	18.00	50.90	12.10	778.00	
Yes	88	36.45	23.65	67.30	12.10	167.00	
**Chest pain**							0.0262
No	428	29.50	18.50	50.40	12.10	778.00	
Yes	94	36.55	21.50	63.40	12.20	265.00	
**Tachycardia**							0.0074
No	440	29.05	18.50	51.50	12.10	778.00	
Yes	82	39.30	21.50	69.70	12.20	178.00	

Supplementary Table 7 Medians and percentiles of IgG plasma levels in relation to symptoms/clinical manifestations

	Positivity ≥12AU/mL						P value*
	N	Median	25°centile	75°centile	Min	Max	
**Pneumonia**							<0.0001
No	493	29.80	18.30	50.20	12.10	778.00	
Yes	29	76.10	58.80	105.00	12.10	178.00	
**Others**							0.1722
No	486	30.40	18.50	52.60	12.10	778.00	
Yes	36	39.65	21.75	58.50	12.10	174.00	
**Number of symptoms/clinical manifestations**							0.014
0 symptoms	62	26.30	18.60	39.70	12.10	257.00	
1 symptoms	56	23.25	17.60	34.50	12.20	778.00	
2 symptoms	65	33.10	16.10	56.60	12.30	160.00	
3 symptoms	61	26.80	18.00	45.40	12.80	128.00	
4 symptoms	55	27.80	19.50	60.50	12.20	265.00	
5 or more symptoms	223	36.60	21.60	61.40	12.10	178.00	
**Fever and Anosmia/Dysgeusia**							<0.0001
No	189	26.10	16.10	48.70	12.10	778.00	
Fever	119	28.80	19.20	44.20	12.20	170.00	
Anosmia/Dysgeusia	80	32.95	21.25	57.85	12.30	174.00	
Fever and Anosmia/Dysgeusia	134	39.10	23.80	63.60	12.10	265.00	
**Total**	522	31.20	18.60	54.80	12.10	778.00	

Kruskal Wallis test statistic for categorical variable, Cuzick’s test for trend for ordinal variables

Supplementary Table 8 Medians and percentiles of IgG plasma levels in relation to comorbidities							
	Positivity ≥12AU/mL						P value*
	N	Median	25°centile	75°centile	Min	Max	
**COPD**							0.3286
No	519	31.30	18.50	55.30	12.10	778.00	
Yes	3	23.40	19.50	24.80	19.50	24.80	
**Asthma**							0.3090
No	491	30.60	18.60	52.70	12.10	778.00	
Yes	31	44.20	17.70	61.40	12.10	158.00	
**Dyslipidemia**							0.2451
No	480	30.40	18.50	52.45	12.10	778.00	
Yes	42	36.25	20.40	65.20	12.30	178.00	
**Active Neoplasia**							na
No	522	31.20	18.60	54.80	12.10	778.00	
Yes	0	.	.	.	.	.	
**History of Neoplasia**							0.0943
No	508	31.40	19.45	55.05	12.10	778.00	
Yes	14	19.30	15.50	35.40	12.50	110.00	
**Chronic hearth failure**							na
No	522	31.20	18.60	54.80	12.10	778.00	
Yes	0	.	.	.	.	.	
**Hypertension**							0.6716
No	477	31.10	18.50	54.80	12.10	778.00	
Yes	45	31.50	19.70	52.10	12.40	174.00	
**History of CHD**							0.1328
No	521	31.20	18.60	53.90	12.10	778.00	
Yes	1	98.40	98.40	98.40	98.40	98.40	
**Atrial fibrillation**							0.7510
No	517	31.20	18.50	54.80	12.10	778.00	
Si	5	20.40	20.40	33.10	19.50	98.40	
**History of TIA/stroke**							0.6328
No	521	31.20	18.60	54.80	12.10	778.00	
Yes	1	23.40	23.40	23.40	23.40	23.40	
**Steatosis/Cirrhosis**							0.0211
No	520	31.25	19.10	55.05	12.10	778.00	
Yes	2	12.50	12.30	12.70	12.30	12.70	
**Other hepatic disorders**							0.5660
No	519	31.20	18.60	55.30	12.10	778.00	
Yes	3	32.80	14.40	35.40	14.40	35.40	

Supplementary Table 8 Medians and percentiles of IgG plasma levels in relation to comorbidities							
	**Positivity ≥12AU/mL**						**P value***
	**N**	**Median**	**25°centile**	**75°centile**	**Min**	**Max**	
**Chronic kidney failure**							0.1197
No	521	31.20	18.60	53.90	12.10	778.00	
Yes	1	110.00	110.00	110.00	110.00	110.00	
**Rheumatoid Arthritis**							0.2702
No	510	31.20	19.20	55.40	12.10	778.00	
Yes	12	31.70	14.60	41.70	13.90	56.10	
**Other immune system disorders**							0.7754
No	483	31.10	18.50	55.40	12.10	778.00	
Yes	39	34.30	20.00	50.10	13.50	147.00	
**Diabetes mellitus**							0.0938
No	521	31.20	18.60	53.90	12.10	778.00	
Yes	1	167.00	167.00	167.00	167.00	167.00	
**Gout**							na
No	522	31.20	18.60	54.80	12.10	778.00	
Yes	0	.	.	.	.	.	
**Other comorbidities**							0.0082
No	482	29.85	18.40	52.00	12.10	778.00	
Yes	40	44.85	26.05	76.15	12.10	139.00	
**Number of comorbidities**							0.2895
0 comorbidity	345	29.10	18.50	52.00	12.20	778.00	
1 comorbidity	133	33.40	19.70	56.20	12.10	178.00	
2 comorbidities	29	30.20	15.70	48.90	12.30	169.00	
3 comorbidities	11	50.10	33.10	65.20	15.50	107.00	
4 or more comorbidities	4	38.25	19.95	83.05	19.50	110.00	
**Total**	522	31.20	18.60	54.80	12.10	778.00	
Kruskal Wallis test statistic for categorical variable, Cuzick's test for trend for ordinal variables							

Supplementary Table 9 Medians and percentiles of IgG plasma levels in relation to vaccinations							
	Positivity ≥12AU/mL						P value*
	N	Median	25°centile	75°centile	Min	Max	
**Influenza vaccine 2019/2020**							0.5250
No	360	31.25	19.25	55.65	12.10	778.00	
Yes	162	30.50	18.50	51.10	12.20	160.00	
**Anti-pneumococcal vaccine**							0.0299
No	500	31.70	19.50	55.65	12.10	778.00	
Yes	22	24.70	15.00	29.40	12.20	139.00	
**Anti-BCG vaccine**							0.6550
No	476	31.25	19.10	53.85	12.10	778.00	
Yes	46	29.25	18.00	56.30	12.10	133.00	
**Other Vaccinations**							0.9761
No	488	31.15	18.50	53.25	12.10	778.00	
Yes	34	32.10	20.30	58.80	12.20	96.60	
**Number of vaccinations**							0.5928
0 vaccination	303	31.50	19.50	55.30	12.10	778.00	
1 vaccination	180	30.30	18.55	53.40	12.10	160.00	
2 vaccinations	33	31.60	14.10	56.60	12.20	139.00	
3 or more vaccinations	6	26.65	18.40	29.40	13.40	45.40	
**Total**	522	31.20	18.60	54.80	12.10	778.00	
Kruskal Wallis test statistic for categorical variable, Cuzick's test for trend for ordinal variables							

S1 Supporting Information. Questionnaire.

Name
Surname
Date of birth
E-mail
Gender
Weight
Height
Cigarettes (n./day)

WORK PLACE

- Humanitas Rozzano (ICH)
- Humanitas San Pio X
- Humanitas Gavazzeni
- Humanitas Mater Domini (HMD)
- Humanitas University (HU)
- Humanitas Medical Care (HMD)

JOB ROLE

- Physician
- Surgeon
- Anesthesiologist
- Nurse
- Radiology Technician
- Lab. Technician
- Biologist
- Physiotherapist
- Staff
- Researcher
- Student
- Other role

Remote working [YES/NO]. If so, indicate how many days per week.

SYMPTOMS/CLINICAL MANIFESTATIONS

Symptoms/clinical manifestations developed since February 1st 2020 to today (first serological test):

- Fever
- Low-grade fever
- Cough
- Sore Throat/Runny nose
- Muscle pain
- Asthenia
- Anosmia/dysgeusia
- Gastrointestinal symptoms (nausea, vomiting and diarrhea)
- Conjunctivitis
- Dyspnea
- Chest pain
- Tachycardia
- Pneumonia (TC confirmation)

COMORBIDITIES

- Chronic obstructive pulmonary disease (COPD)
- Asthma
- Dyslipidemia/High Cholesterol (high blood cholesterol/triglycerides)
- Active Neoplasia (NPL)
- History of Neoplasia
- Hypertension
- Cronic hearth failure
- History of coronary heart disease (CHD)
- Atrial fibrillation
- History of TIA/stroke
- Chronic kidney failure
- Steatosis/Cirrhosis
- Other hepatic diseases
- Rheumatoid Arthritis
- Other Immune system diseases
- Diabetes mellitus
- Gout
- Other comorbidities

VACCINATIONS

Influenza vaccine 2019/2020 [YES/NO]
Anti-pneumococcal vaccine[YES/NO]
Anti-TBC vaccine [YES/NO]
Other vaccines

SARS-CoV-2 EXPOSURE

Have you been diagnosed with COVID-19? [YES/NO]

If so, when? (indicate the month)

Have you had interactions with people affected by COVID-19? [YES/NO]

If so, who was it?

- Colleague
- COVID-19 patient
- Family member
- Other
